# Transition to Adulthood in Spina Bifida: Changing Roles and Expectations

**DOI:** 10.1100/tsw.2007.179

**Published:** 2007-11-26

**Authors:** Shubhra Mukherjee

**Affiliations:** Pediatric and Adolescent Rehabilitation Medicine, Rehabilitation Institute of Chicago and Children's Memorial Hospital Spina Bifida Clinic, Northwestern University Feinberg School of Medicine, Chicago, IL, USA

**Keywords:** transition, Spina Bifida, adulthood, adolescence

## Abstract

Survival to adulthood for people with Spina Bifida now exceeds 85% due to improvements in medical and surgical management. Rates remain lower than expected for community participation, healthy lifestyle choices, employment and independent living. The importance of transition programming to help adolescents with disabilities prepare for adult life roles is now understood. Literature currently is mainly conceptual or descriptive, but informs the process of developing transition program models. The need for competent and effective adult care providers is discussed. Both the transition to adulthood and the transfer of care to adult care clinics are important and distinct components of spina bifida lifespan care.

## INTRODUCTION

Adults with spina bifida are an increasing population, thanks to the success of medical and surgical management for hydrocephalus, back closure, and neurogenic bladder and bowel. Antibiotic use after WWII, introduction of the VP shunt in the 1950s, and intermittent catheterization in 1970 have dramatically reduced mortality in spina bifida. Survival to adulthood is now over 85-90% for people born with spina bifida[1]. Advancements in the field of rehabilitation, mobility aids, accessibility and technology have changed what is possible for a person with a significant disability. Legal mandates, policy, and increased social expectations have resulted in great progress towards optimal integration for all people with disabilities over the last 40 to 50 years. However, community and society participation are still limited for many adults with spina bifida. Issues of transition to adulthood, and barriers to transfer of care to adult providers have become widely identified as contributing factors[2].

Transition has been defined as the "purposeful, planned movement of adolescents and young adults with chronic physical and medical conditions from child-centered to adult-oriented health-care systems[3]." The idea of transition to adulthood and to adult care consists of 2 major themes. The first is the preparation for adult life roles and self-determination. Increasing experiences and gradually increased responsibility help this to occur. The second aspect is transfer of care to adult health care providers, sometimes moving to an adult care facility. A study by Scal demonstrated that only 50% of youth with special health care needs had discussed transfer to adult care[4]. A study by Reid in congenital heart condition patients showed the 47% had transferred care successfully to adult specialty clinics[5]. Although there has been progress in this arena, there is much that remains to be done.

Similar transition themes have been recognized for other groups with chronic health conditions, where survival has improved into adulthood. Sickle cell disease, diabetes, cystic fibrosis, cerebral palsy, congenital heart disease and renal disease are examples[6,7,8,9]. Spina bifida is one of the most complex of congenital conditions resulting in physical disability, and affects multiple body systems. The process of transition from adolescence to adulthood is a complex one. Managing a chronic health condition can further complicate the process. Ensuring that teens with chronic conditions gain the skills necessary to succeed in adult life roles as independently as possible requires fostering and attention, providing experiences, encouragement and advice.

Developmental tasks of typical adolescence have been described by various authors, and are summarized here:

I. Development of a self-identity (values, personality, sexual identity)
II. Reduced dependence on parents/caregivers (physical, emotional, financial)
III. Development of social relationships (via appropriate behavior and communication skills) establishing a vocation or meaningful way to spend time
IV. Establishing a vocation or meaningful way to spend time (hobby, volunteering, child care)

Using this framework, incremental skills in each area can be developed using various experiences. Physical and occupational therapists are needed again during this stage to assess these skills, and provide training specific to the impairments and goals, such skills include transfers, dressing, and budgeting. If the long-term reality is likely to be dependence, due to physical or cognitive impairment, then long term planning will be required to provide for those needs. Assisted living, guardianship or estate planning may be required in those cases.

Changes to the legal rights of the person with a disability such as 1973 Rehabilitation Act, the ADA (Americans with Disability Act) in 1990, and IDEA (Individuals with Disability Education Act) in 1975 have increased integration and expectations by and for people with disabilities for community participation. High profile role models have also led the charge for active participation, including disability rights activists Justin Dart and Bob Kafka, athlete Rick Hansen, Vancouver mayor Sam Sullivan, and actor Christopher Reeve. The importance of role models and mentors is being recognized and used through various organizations, including Spina Bifida Association (SBA). This provides young people with disabilities a sense of possibility and success, which comes from realizing that someone “just like me” has become a leader.

## TRANSITION LITERATURE

Medical literature on transition to adulthood with chronic childhood-onset conditions is increasing, but primarily in the pediatric literature. Medical professional bodies have published policy or guidelines on transition to adulthood, such as the America Academy of Pediatrics (AAP), along with AAFP and ASIM[10], and the Society for Adolescent Medicine[11]. Slowly, the same themes are beginning to enter the adult medical literature as well.

Principles of transition have been published by various groups, include the following:

1. Transition is a process, not an event.
2. The transition process should start as early as possible.
3. Transition is individual centered, and includes the family.
4. Transition should comprise developmentally appropriate care.
5. Transition planning is flexible and future oriented.

A review of the literature reveals that it is primarily conceptual or descriptive thus far. Descriptions of current status of adults with pediatric onset disabilities are available from a variety of countries[12,13,14]. The findings are concerning. According to CDC data, health is poorer, and rates of depression, obesity and smoking are higher than those without disabilities. Rates of employment are low at 25-54%, many young adults continue to live with their parents, and few are married[15,16]. There is social isolation, and poor physical activity and community participation. However, quality of life is often rated as high[17,18]. Cognitive issues for people with spina bifida have been described, and include memory, processing speed, problem solving[19,20]. Verbal skills are relatively preserved[21,22]. Vocational counseling has been one key area that families request assistance in[23]. Exactly how the cognitive issues impact development of adult life skills is slowly being described. Transition research may help clarify what components can be ameliorated by experience and training, and which areas will need to be compensated for in other ways, by personnel or assistive devices.

Checklists and guidebooks on how to facilitate development of adult life skills are available through many websites and publications (www.hrtw.org/tools, http://depts.washington.edu/healthtr/). A recent review of 5 review articles on transition showed similar themes: a need for increased knowledge on best practices[24]. One study attempted to track a group of 10 young adults as they were transferred to adult care providers, and documents their experience with the process. Barriers and dissatisfaction were due to less consistent providers, and fear of less regular or less attentive care[25]. To date, no study published has shown results of instituting a transition program, demonstrating improved outcomes compared to baseline or to an untreated cohort.

Studies have demonstrated associations of successful transition to adulthood with having chores, having supportive family and a network of friends[26]. People with spina bifida have been described as having features of being passive and socially immature[27]. This author has found anecdotally that the passivity is more apt to change in early teens. By the mid twenties, the passivity appears to be a fixed behavior, and appears less amenable to change. This would deem transition programming for self-determination and active participation, to be a critical area of focus during the junior high and high school years. Once passive behaviors are set, it is very difficult to change them in adulthood. Passivity seems to be linked with not taking responsibility, which could lead to less optimal care and perhaps worse health outcomes. Whether this passivity is learned behavior due to overprotective parents and caregivers, or a cognitive issue is not known.

Outcomes of successful transition need to be defined. Goals of transition programs are to improve community participation, including increased employment, increased community living, and increased time spent in community and social activity. Maintenance of health, and reduced preventable secondary conditions is desirable. Ideally, daily care and health maintenance should not be excessively time-consuming. Costs of programs and cost-benefit of programs delivered needs to be understood better. One stated goal of transition services is to improve quality of life. This is difficult to measure, and can be defined as satisfaction with life, freedom from pain or depression, or freedom from impairments. It is defined formally as the ability to enjoy normal life activities. Quality of life has been shown to correlate to reduced life stress, adequate social supports, and community participation, rather than impairment[28,29,30]. Ongoing assessment of quality of life into adulthood will be important as an outcome measure for adult care.

Health issues in adulthood continue to occur. These include pressure sores, shunt issues, syringomyelia, tethered cord, urologic issues, bowel issues, lymphedema, fractures, etc. Serious complications such as renal deterioration, osteomyelitis and amputation can be preventable, with proper care and education[31]. At present many centers continue to care for people with spina bifida when they are adults, in pediatric care centers.

## TRANSFER OF CARE TO ADULT PROVIDERS

The importance of proper adult oriented care is becoming apparent. A paper comparing outcomes of adults after a clinic disbanded demonstrated increased preventable surgeries, when there was no person responsible for coordinating the care of the patient[31]. Personal care independence is shown to be adversely affected by increased number of shunt malfunctions[12,22]. Quality age-appropriate health care is often not accessible for adults, similar to that which kept them healthy until adulthood.

Adult health care settings often have less support staff, social work and clinic coordinators, where the individual is expected to be responsible. Studies have demonstrated that costs in adult care hospitals are lower, and lengths of stay are sometimes lower for people with childhood onset conditions such as sickle cell and spina bifida[32]. Leadership from adult care providers needs to be developed. It is lacking due to inadequate knowledge or training in pediatric onset conditions, poor reimbursement, frustration with less responsible patients, and less support staff for coordination of complex care.

Transfer of care to adult providers is an important marker of success of transition. This seems most successful when there is a period of shared care with the pediatric team, either in joint clinics, or concurrent care. Information transfer is extremely important, such as how a patient presents with shunt malfunction, especially if it is atypical. The loss of the close relationships with trusted pediatric care providers is difficult for many families. This process takes time to become comfortable with, and to gain trust in the new providers. The cultural shift of increased responsibility, and individual versus family centered care is also difficult, but demonstrates the ability of the patient to succeed in a life-skill responsibly. The young adult should be able to identify who is managing the medical care, and which medical provider to contact if they experience problems. Transfer of health care to adult care providers has advantages, including being age-appropriate, lower cost[32], and may be safer. Adult health providers have been slow to accept responsibility for establishing protocols or special clinics for these transferring patients. Where such adult clinics have developed, clinicians say that the adult outcomes have influenced pediatric care positively.

## CONCLUSION

Preparing youth for adult roles is the goal of transition programming. Slow advancements have been made over 20 years since the Surgeon General’s conference in 1989[33], which need to increase in scope and speed. Individualized attention to help youth prepare for adult roles appears to be the key. Transition Coordinator roles are being adopted at a few clinics and rehabilitation centers to provide this important guidance[34]. Online and paper resources have assisted proactive parents and care providers with transition skills preparation, and now need to be delivered consistently to all children who are traversing this divide between adolescence and adulthood. More work is needed to make all pediatric care providers aware of how to foster responsibility and self-determination for youth with special health care needs.

There are still issues with social discrimination, access to affordable health insurance, transportation that is efficient and affordable, and access to employment that is flexible to health needs. Access to fitness opportunities, primary health care, and other amenities also need to be improved for optimal outcomes. Progress is being seen in cities throughout the US in some of these domains, often through efforts of disability advocacy organizations.

Preparing well adjusted, active, healthy young adults for adult health care in age-appropriate settings, will lead to greater health and quality of life outcomes. Healthy aging through good lifestyle habits such as exercise, healthy eating and controlling obesity, as well as preventative and proactive health care will continue to lead to improved health and longevity for adults with spina bifida. Pediatric providers will need to partner with adult providers to promote transfer of care once patients are ready. Currently evidence is needed in the areas of demonstrating best practices and improved outcomes from transition programs. Expertise in managing adult health issues and complications has already shown signs of influencing pediatric care. Seamless transition through information transfer and pediatric team to adult team communication will be vital to the success of transfer of care. Adults with spina bifida deserve no less than full attention of adult care providers who can assist them in living full active lives, and remaining healthy as they age, so they too can pursue all their goals and dreams.
